# Effect of Bergenin on Human Gingival Fibroblast Response on Zirconia Implant Surfaces: An In Vitro Study

**DOI:** 10.3390/jfb14090474

**Published:** 2023-09-15

**Authors:** John Xiong, Catherine M. Miller, Dileep Sharma

**Affiliations:** 1 College of Medicine and Dentistry, James Cook University, Smithfield, QLD 4878, Australia; john.xiong@my.jcu.edu.au (J.X.); kate.miller1@jcu.edu.au (C.M.M.); 2 School of Health Sciences, College of Health, Medicine and Wellbeing, The University of Newcastle, Ourimbah, NSW 2258, Australia

**Keywords:** zirconia, dental implant, fibroblasts, bergenin

## Abstract

The poor quality of life associated with the loss of teeth can be improved by the placing of dental implants. However, successful implantation relies on integration with soft tissues or peri-implant inflammatory disease that can lead to the loss of the implant. Pharmacological agents, such as antibiotics and antiseptics, can be used as adjunct therapies to facilitate osseointegration; however, they can have a detrimental effect on cells, and resistance is an issue. Alternative treatments are needed. Hence, this study aimed to examine the safety profile of bergenin (at 2.5 μM and 5 μM), a traditional medicine, towards human gingival fibroblasts cultured on acid-etched zirconia implant surfaces. Cellular responses were analysed using SEM, resazurin assay, and scratch wound healing assay. Qualitative assessment was conducted for morphology (day 1) and attachment (early and delayed), and quantitative evaluation for proliferation (day 1, 3, 5 and 7), and migration (0 h, 6 h and 24 h). The concentrations of bergenin at 2.5 μM and 5 μM did not demonstrate a statistically significant effect with regard to any of the cellular responses (*p* > 0.05) tested. In conclusion, bergenin is non-cytotoxic and is potentially safe to be used as a local pharmacological agent for the management of peri-implant inflammatory diseases.

## 1. Introduction

Dental implantology is a predictable, clinically proven method to restore edentulous areas to improve form, function and aesthetics [1]. Since its introduction, titanium and its alloys have been regarded as the gold standard implant material, with well-established clinical results due to its excellent biocompatibility and mechanical strength [2,3]. The principal disadvantage of titanium implants is its unaesthetic metallic appearance, which is often visible through peri-implant mucosa, particularly in patients with a thin mucosal biotype [4,5]. Titanium has also been reported to induce potential immunological complications due to the release of titanium ions into the surrounding structures, resulting in implant failure [6]. To combat this, novel technologies have been explored. Zirconia has been recommended due to its toothlike colour, low affinity for plaque and outstanding mechanical and chemical properties [7,8,9]. Zirconia is a bioinert non-resorbable metal oxide that has demonstrated excellent mechanical and chemical properties and offers a variety of potential advantages for use in implant dentistry, especially in the aesthetic zones [7,10,11].

The formation of an early and long-standing soft tissue barrier is imperative for both the initial healing and increasing the longevity of the implant restoration [12,13]. Human gingival fibroblasts are the soft tissue cells involved in forming the tight soft tissue adaptation against the implant neck. Additionally, they are involved in the synthesis and maintenance of the extracellular matrix, which is responsible for facilitating tissue regeneration and repair during wound healing [14]. The first phase of soft tissue healing involves cellular attachment, which serves as a basis for subsequent cellular interactions, such as proliferation and spreading [15]. The tight soft tissue adaptation onto the implant neck is made up of a complex with epithelium and connective tissue cells, however, unlike natural teeth, it lacks periodontal ligaments. This makes the implant more prone to bacterial penetration and epithelial downgrowth, causing bone loss and, ultimately, the loss of the implant [16,17]. Hence, this soft tissue barrier is of paramount importance in ensuring a soft tissue seal that is essential for preventing microbial ingrowth and maintaining successful osseointegration.

The 2017 World Workshop Classification of Periodontal and Peri-implant Disease and Condition outlines the two disease processes associated with the peri-implant tissue: peri-implant mucositis and peri-implantitis [18]. Peri-implant mucositis is limited to the mucosa of the implant and is characterised by the redness, swelling and inflammation of the peripheral soft tissue [17,18,19]. It is reversible with proper routine implant maintenance and good oral hygiene; however, if untreated, it will progress to peri-implantitis [17,18,19]. Peri-implantitis is an infectious inflammatory disease associated with the loss of surrounding peri-implant bone and tissue, and is one of the main causes of implant failure [19].

Various approaches have been developed for the clinical management of peri-implantitis. The physical methods of decontamination are routinely practiced; however, such strategies can damage implant surfaces and predispose them to bacterial colonisation [9,20]. Adjunct therapies using chemical and pharmacological agents have been employed to aid in the management of mucositis and peri-implantitis through the decontamination of the implant surface and may include antibiotics and antiseptics [21,22,23]. Several studies have demonstrated a negative impact on the cellular proliferation of commonly used oral antiseptics (i.e., CHX, Povidone-Iodine and Hydrogen peroxide) and antibiotics that are commonly prescribed for management of peri-implant infections [24,25,26,27,28,29]. These results, coupled with the rise of resistance to commonly used antiseptics and antibiotics, makes it imperative that alternative treatments be identified. 

Natural immunomodulatory agents, especially derived from plants, have been extensively used in folk medicine as nootropics and adaptogens for centuries [30]. Recently, bergenin, which is derived from plants of the *Bergenia* genus, has been shown to have favourable biological activities that may be beneficial in wound healing [31,32,33]. Current research suggests that bergenin possesses anti-inflammatory properties through the reduction in cyclooxygenase-2 activity (COX-2), the inhibition of pro-inflammatory cytokines, such as interleukin-6 (IL-6) and IL-8, and the selective inhibition of COX-2 in vitro [32,34]. Additionally, bergenin has been reported to possess anti-microbial activities against *Candida albicans* and Herpes simplex virus [32]. In the context of its effect on bone, a study reported that bergenin may enhance osteoblastic bone regeneration [35]. Hence, bergenin could potentially be used as a novel therapy for managing peri-implant inflammatory diseases via local delivery, however, its effect on soft tissue cells remains unexplored. Based on the known actions of bergenin, we hypothesise that the presence of bergenin could have no negative effect on the gingival fibroblasts cultured on the zirconia surfaces. Hence, our study aims to explore the effect of bergenin on HGF cells on the attachment, proliferation and migration on Zirconia implant surfaces.

## 2. Materials and Methods

All cellular assays were completed in accordance with the Minimum Information About a Cellular Assay (MIACA) guidelines [36]. The Modified Consolidated Standards of Reporting Trials guidelines for preclinical in vitro studies on dental materials checklist was used to report the findings [37]. No ethics approval was required for this in vitro study.

### 2.1. Sample Preparation

The yttria-tetragonal zirconia polycrystals (Y-TZP) discs were kindly provided by Dr. Elsa Dos Santos Antunes (James Cook University) and were produced via the sintering of 3 mol% yttria partially stabilised zirconia powder (30% monoclinic and 70% tetragonal) using the protocol described in Munro et al. (2020) [8]. The discs measuring 14 mm in diameter and 1 mm in thickness were utilised in this study, and surface modification was performed as previously reported [9]. Briefly, discs were modified by submersion in 40% hydrofluoric acid (Scharlab, Barcelona, Spain) for 1 h to create an acid-etched surface (AEY-TZP). Following this, the discs were rinsed with deionised water to remove any residue and to neutralise any remaining acid and were autoclaved prior to cell culture.

#### Complete Media Preparation

The complete media (CM) was made up of Dulbecco’s modified Eagle medium (DMEM) supplemented with 10% foetal bovine serum (FBS) (Sigma-Aldrich, Sydney, Australia), penicillin/streptomycin (Sigma-Aldrich, Sydney, Australia) and L-glutamine (Sigma-Aldrich, Sydney, Australia). Bergenin (Sigma-Aldrich, Sydney, Australia) was titrated into the CM at concentrations of 0 μM (B0), 2.5 μM (B2.5) and 5 μM (B5). The complete media containing bergenin is referred to as BCM. 

### 2.2. Cell Culture

Commercially sourced Human gingival fibroblasts (HGF; ATCC^®^ PCS-201-018™) were used in this study. HGF cells were grown in cell culture flasks with CM. The cells were incubated at 37 °C, 5% CO_2_ and 90% humidity, with the media being changed every 3 days. Cells were checked under conventional microscopy (Nikon EclipseTS100, Nikon Instruments, Tokyo, Japan) until 95% confluency and were then passaged. Cells from the 3rd to 7th passage were used. The cells were then detached with trypsin (Sigma-Aldrich, Sydney, Australia) and seeded onto the AEY-TZP discs or tissue culture plates with CM containing differing concentrations of bergenin for their relevant experiment. 

#### 2.2.1. Cell Morphology and Attachment

A qualitative analysis was performed to observe the cytoskeletal arrangements of the HGF cells on AEY-TZP discs using Scanning electron Microscopy (SEM) (Phenom™ G2 pro, Phenom-World BV, Eindhoven, The Netherlands). Initially, HGF (3 × 10^5^ per disc) were seeded onto the AEY-TZP discs incubated in BCM (0 μM, 2.5 μM or 5 μM) for 24 h in triplicate. Cells were fixed with 3% glutaraldehyde in 0.1 M phosphate-buffered saline (PBS; Sigma-Aldrich, Sydney, Australia), rinsed with 0.1 M PBS twice, then dehydrated in an increasing ethanol series (25%, 50%, 75%, 95%, then 100%) for 5 min at each concentration. Following this, each disc was dried in a 1:1 solution of hexamethyldisilane (HMDS) and ethanol for 15 min, and 100% HMDS for 5 min. Prior to metallising, samples were dried in a fume cupboard for 4 h. Gold sputtering was performed on the samples and observed by SEM at 500×, 2000×, and 5000× magnification. Cell morphology was assessed on micrographs taken in triplicate randomly in different areas of each experimental disc.

Cellular attachment was assessed at an early and late timepoint. HGFs at a density of 3 × 10^5^ cells/well were seeded onto the AEY-TZP surfaces (n = 9) in a 24-well plate in BCM (0 μM, 2.5 μM or 5 μM). After culturing for 30 min (early) or 3 days (late), unattached cells were removed by rinsing three times with 1 mL of PBS. The attached cells were fixed with 4% formaldehyde at room temperature for 10 min. Following this, cells were permeabilised in 0.1% Triton X-100 (Sigma-Aldrich, Sydney, Australia) for 10 min and then blocked with 1% bovine serum albumin (Sigma, St. Louis, MO, USA) for 15 min to prevent non-specific binding. After rinsing with PBS (3 × 5 min each), the cells on the discs were stained with 2% Flash Phalloidin™ red solution (BioLegend, San Diego, CA, USA) according to the manufacturer’s instructions. Fluorescence was visualised and imaged using an Olympus IX53 inverted epifluorescence microscope (Evident Australia Pty Ltd., Macquarie Park, Ryde, Australia).

#### 2.2.2. Cell Proliferation

Human gingival fibroblast proliferation on the surface of the specimens was evaluated using the resazurin assay. The HGF cells were seeded onto the AEY-TZP surfaces (n = 9) as follows: On day 0, 400 µL of BCM (0 μM, 2.5 μM or 5 μM) containing 1 × 10^5^ cells/mL was placed to seed cells onto each surface. Cellular proliferation was determined at days 1, 3, 5 and 7 using 10% *v*/*v* resazurin (Sigma-Aldrich, Sydney, Australia). The resazurin solution was added at each time point and incubated for 5 h. Media samples from each specimen were transferred to a 96-well plate in triplicate (3 wells of 100 µL each). The absorbance of resorufin (the product of reduction) at 570 and 600 nm was read using a microplate absorbance reader (iMark™ Microplate Absorbance Reader, BioRad Laboratories, Hercules, CA, USA). The percentage of resorufin was calculated using the values obtained for the control solution (TCP + resazurin solution without cells).

#### 2.2.3. Cell Migration

A scratch-healing assay was used to assess migration. HGF cells (3 × 10^5^ cells/mL) were seeded onto three groups of AEY-TZP (n = 3) surfaces and TCP (n = 3) in a 24-well plate with 400 µL of CM. The cells were then incubated at 37 °C, 5% CO_2_ and 90% humidity until confluent. Prior to initial scratching, the CM was changed to BCM for the respective groups (0 μM, 2.5 μM or 5 μM). Using a 200 µL pipette tip, two scratches were made perpendicular to each other on the discs and TCP. Following this, detached cells were removed by washing each well thoroughly with PBS. The migration of cells into the scratched area was assessed at 0 h, 6 h and 24 h. Prior to imaging, samples were washed in 0.1% Triton X-100 for 10 min, then blocked with 1% bovine serum albumin (Sigma, St. Louis, MO, USA) for 15 min. Following that, surfaces were rinsed thrice with PBS for 5 min each time. The discs and wells were stained with 2% Flash Phalloidin™ red solution (BioLegend, San Diego, CA, USA) according to the manufacturer’s instructions. The scratched area was imaged using an Olympus IX53 inverted epifluorescence microscope (Olympus Australia Pty Ltd, Melbourne, Australia) at 10× magnification. The wound area was measured using the Fiji app on ImageJ software (version 1.53f51, National Institutes of Health, Bethesda, MD, USA), and the healed area was calculated by comparing the wound area at a set time point to the initial scratch area and represented as a percentage. The percentage calculation is shown below and was quantified using the measured scratched area (SA_Measured_) and the average initial scratch area (SA_Initial_). Measurements were taken in triplicate, and the average surface area was used for the calculations. For all groups initial (0 h) healed percentage was 0%.

$$Healed\;Percentage\;\left(\%\right)=\frac{{SA}_{Initial}{-SA}_{Measured}}{{SA}_{Initial}}\times 100\%$$

### 2.3. Statistical Analysis

The software suite GraphPad 9.2 (GraphPad Software, San Diego, CA, USA) was used for statistical analysis. Two-way ANOVA and post hoc Tukey test were used to compare the control and the experimental groups since the data did not show significant departure from normality. The results were expressed as mean ± standard deviation. A *p* < 0.05 was considered statistically significant.

## 3. Results

### 3.1. Cellular Morphology

**Figure 1:**
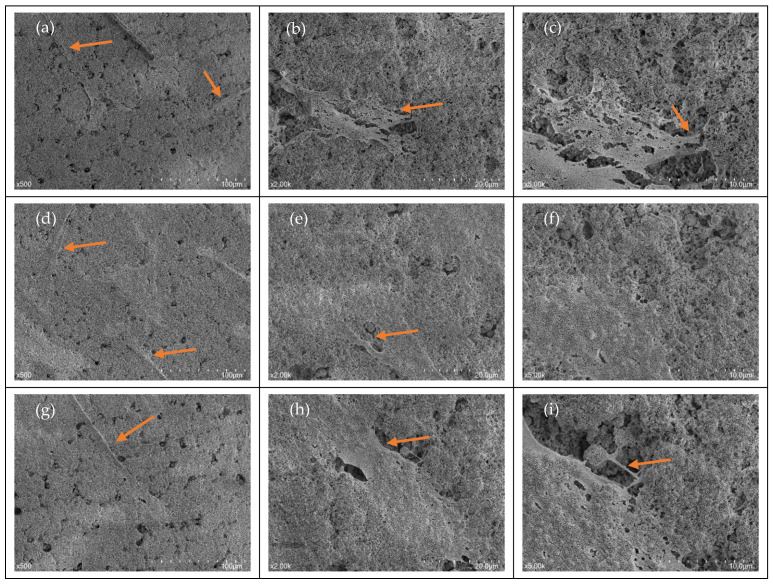
SEM Imaging of HGF morphology AEY-TZP discs (**a**–**i**) (bergenin 0 μM, (**a**–**c**), bergenin 2.5 μM, (**d**–**f**), bergenin 5 μM, (**g**–**i**)). Cells were fixed with 3% glutaraldehyde, discs were dried then gold sputtered, and SEM imaging was performed on three randomly selected sites. These images are representative and portray definitive the morphology of spindle-like long cytoplasmic elongations or pseudopods (orange arrows), narrow and flattened profiles. Scale bar = (**a**–**c**) 100 μm, (**d**–**f**) 20 μm, (**g**–**i**) 10 μm. SEM images taken at 500×, 2000×, 5000× magnification after 24 h.

An SEM analysis of cellular morphology was performed and aimed to identify the characteristics of fibroblasts upon initial attachment. Following an incubation period of 24 h, cells were found to be adhering to both the TCP and AEY-TZP discs for all bergenin concentrations (Figure 1). SEM observation showed the definitive morphology of spindle-like long cytoplasmic elongations, a profile characteristic of HGFs. 

#### Cellular Attachment

**Figure 2:**
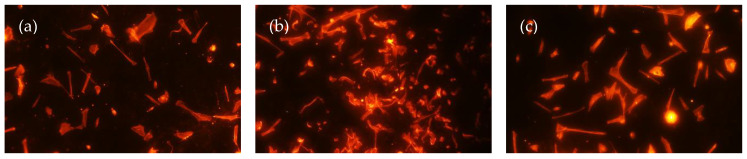

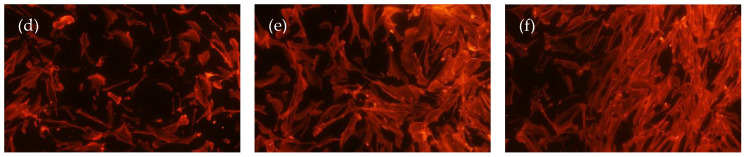
Cellular attachment of HGF cells on AEY-TZP discs (10×). The results show an increase in cell numbers within the groups with CM containing Bergenin. Cells were imaged on each sample using Flash Phalloidin™ Red solution and epifluorescence microscopy. Representative images of the attachment are shown: early attachment (**a**–**c**) (bergenin 0 μM (**a**), bergenin 2.5 μM, (**b**), bergenin 5 μM (**c**)), delayed attachment (**d**–**f**) (bergenin 0 μM (**d**), bergenin 2.5 μM, (**e**), bergenin 5 μM, (**f**)).

The immunofluorescence imaging aimed to observe patterns of the cellular attachment of HGF onto AEY-TZP discs in the presence of differing concentrations of bergenin. Both early and delayed attachment were observed and are shown in Figure 2. During early attachment, there was no observable difference in cellular morphology or cell volume between the groups. However, in the delayed attachment phase, there was a noticeable increase in cell numbers in the presence of bergenin compared to untreated cells, independent of bergenin concentration. Additionally, the cells demonstrated a similar morphology identified in the SEM analysis with random orientation. This indicated that bergenin had minimal to no negative effect on the attachment of HGF onto AEY-TZP discs.

### 3.2. Cellular Proliferation

**Figure 3:**
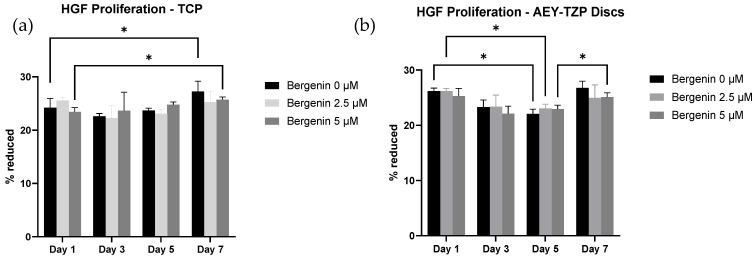
Cellular proliferation of HGF was observed by assessing the average percentage of reduction in resazurin by the cells cultured onto TCP (**a**) and AEY-TZP (**b**) discs in wells containing 400 μL of CM and BCM. The assay used 10% *v/v* resazurin to determine cellular proliferation at day 1, 3, 5 and 7. Results are plotted as mean ± standard deviation. The results demonstrated that bergenin had minimal effect on the rate of cellular proliferation (*p* > 0.05). * indicates a significant difference (*p* < 0.05) between two groups.

The resazurin assay aimed to investigate the effects of bergenin on cellular proliferation. Results at 1, 3, 5 and 7 days of incubation are shown in Figure 3. Post hoc analyses demonstrated a statistically significant effect associated with time (F (1.561, 9.368) = 6.724, *p* < 0.05) for both AEY-TZP discs and TCP, with cell numbers increasing over time. For TCP, there was a significant increase in cellular proliferation between day 1 and day 7 (*p* < 0.05) for the untreated cells and cells treated with bergenin, with no significant difference between groups (Figure 3a). For cells grown on AEY-TZP discs, 2.5 μM bergenin-treated samples showed a decrease in proliferation from day 1 to day 5, whilst cells treated with 5 μM bergenin had a significant increase in proliferation from day 5 to day 7 (Figure 3b). However, there was no significant effect of differing bergenin concentrations over time (F (6, 18) = 1.368, *p* > 0.05). This assay demonstrated that the tested concentrations of bergenin did not affect cellular proliferation at any time point sampled.

### 3.3. Cellular Migration

**Figure 4:**
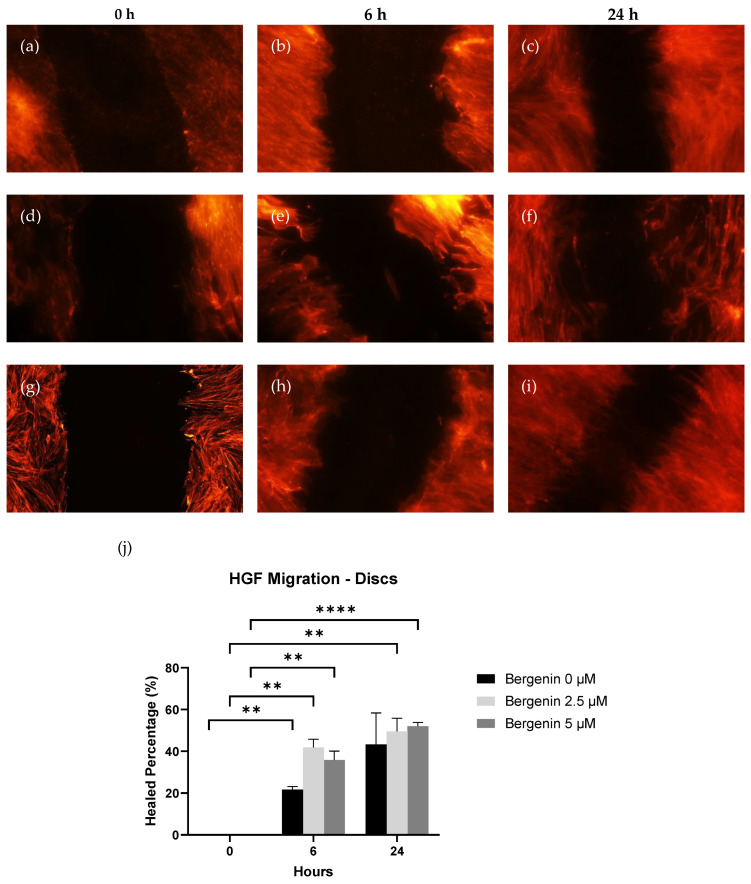
Cellular migration of HGF on AEY-TZP discs was observed by calculating the average healed percentage across time points. HGF cells were seeded onto AEY-TZP Discs in wells containing 400 μL of CM and grown until confluent. Prior to the initial scratch, the BCM media was allocated into the wells. Following the scratch, 2% Flash Phalloidin™ red solution was used, and images were observed under epifluorescence microscopy. Representative images (10×) of the migration were chosen: bergenin 0 μM (**a**–**c**), bergenin 2.5 μM, (**d**–**f**), bergenin 5 μM, (**g**–**i**), 0 h (**a**,**d**,**g**), 6 h (**b**,**e**,**h**), 24 h (**c**,**f**,**i**). The images were analysed using ImageJ software, and the healed percentage was calculated and compared (**j**). The cells grown in 0 μM bergenin showed a significant increase in healed percentage at 6 h compared with 0 h but no significant increase from 0 h to 24 h. The presence of bergenin did not have a significant effect on healed percentage across the time points compared to 0 μM bergenin (*p* > 0.05) ** indicates a *p* < 0.01, **** indicates a *p* < 0.0001.

The scratch wound healing assay aimed to observe any differences in healing when cells were cultured in the presence of bergenin following an initial scratch using a 200 µm pipette tip. Results can be seen in Figure 4. The average surface area (S_a_) for each scratch at 0 h was 0.5379 mm^2^ for AEY-TZP discs. 

At 6 h, all groups (B0, B2.5, B5) demonstrated a significant increase in healed percentage (24.8%, 41.9% and 32.7%, *p* < 0.05) compared to 0 h. At 24 h, B2.5 (49.5%) and B5 (52%) showed a significant increase compared to 0 h; however, no groups demonstrated a significant increase compared to 6 h. Within the groups, only B2.5 demonstrated a significant increase over B0 (*p* < 0.05) for AEY-TZP discs, all other experimental groups did not show significant differences to each other. A comparison of the differing concentrations of bergenin showed that at 6 h, the healed percentage of B2.5 (41.9%) was significantly larger (*p* < 0.05) than B0 (24.8%). Apart from this, no other groups demonstrated any significant difference between the groups at both timepoints (6 h, 24 h).

The results demonstrate that, although a greater final healed percentage was noted at 24 h in the cells cultured with bergenin on the AEY-TZP discs, bergenin did not have a significant effect on the cellular migration of HGF cells. 

## 4. Discussion

A biological seal of soft tissue around the transmucosal component of the dental implant is very important for long-term stability. The lack of a proper seal allows for the penetration of bacteria and the stimulation of an inflammatory response that can compromise the integrity of the implant. We hypothesised that the addition of a molecule that has anti-inflammatory as well as anti-microbial effects may enhance soft tissue healing and promote osseointegration. Our results provide insight into the effect of bergenin on the attachment of HGF on zirconia implant surfaces. The potential biological effects were assessed using SEM to assess the morphology and attachment, a resazurin assay to assess subsequent proliferation, and a scratch wound healing assay to assess migration across the surface. The assays were performed to establish a safety profile of bergenin in the context of HGF cells and to identify its ability to promote healing. Our results demonstrate that bergenin-exposed cells exhibited normal morphological characteristics upon attachment and exposure did not impede cellular proliferation, supporting our hypothesis that bergenin had no negative effects on cells. It is important to note that successful wound healing is complicated and relies on many different physiological factors, such as inflammation, and thus cannot be determined by the cellular activity alone. We hypothesized that the addition of a molecule that has anti-inflammatory as well as anti-microbial effects may enhance soft tissue healing and promote osseointegration. Thus, adding bergenin, which has been shown to have anti-inflammatory actions in inhibiting pro-inflammatory cytokines, such as IL-6 and IL-8, as well as anti-microbial actions, may foster an ideal environment for healing [32].

SEM analysis demonstrated that HGF demonstrated similar morphology amongst tested groups. They presented with narrow spindle-like shape with long cellular extensions indicative of optimal migration and attachment capability [38]. The cells demonstrated similar morphology to those reported in a study by Zizzari et al. (2013), which examined HGF attachment on machined and polished Y-TZP discs following 3 h, 24 h, 72 h and 7 days of incubation. They reported that at 24 h and beyond, both surfaces demonstrated HGFs with definitive morphology and long cytoplasmic elongations, consistent with what was observed on acid-etched discs in this present study [39]. In the current study, acid-etching on the Y-TZP discs was performed as it is a common surface modification technique intended to enhance osseointegration [40]. Whilst surface roughness parameters were not recorded in this study, it has been noted across the literature that surface structure can affect cellular morphology and adhesion [41,42]. The current literature regarding the effect of the roughness of the Zirconia surface on adhesion is varied, but parallel grooves are known to promote elongated morphology and can orientate HGF following surface morphology [41,43]. Our study demonstrated that bergenin did not affect the morphology or adhesion ability of HGF cells, suggesting that bergenin did not alter the initial cellular attachment of human gingival fibroblasts or negatively affect morphology. With the immunofluorescence imaging, it was noted visually that there was a greater volume of cells in the bergenin-exposed groups compared to unexposed cells.

The resazaurin assay for cellular proliferation revealed that bergenin also did not have a significant effect on the rate of cellular proliferation. To date, there is no literature that explores the effect of antiseptics and antibiotics on HGF proliferation in the context of zirconia surfaces; however, several studies demonstrate the negative impact of commonly used oral antiseptics (i.e., CHX, Povidone-Iodine and Hydrogen peroxide) on cellular proliferation and the negative impact of antibiotics that are commonly prescribed for the management of peri-implant infections [24,25,26,27,28,29]. Emmadi et al. (2008) noted the dose-dependent effect of oral antiseptics, with CHX (0.2%) being more cytotoxic than Povidone-Iodine (1%) [24]. Similarly, other studies by Wilken et al. (2001) and Cline and Layman (1992) confirmed that the direct exposure of between 0.0025% to 0.12% CHX and 0.2% Povidone-Iodine was enough to cause the inhibition of growth. [27,28] The findings of this current study demonstrate that bergenin exposure does not affect the proliferation of HGF cells and may suggest the neutral effect on cell growth within the early stages of healing.

Previous animal and clinical studies [44,45] showed that a faster gap closure between soft and hard tissue around the abutment can facilitate a healthy peri-mucosal tissue; therefore, an increase in cell density of HGF could promote wound healing after insertion. In the scratch wound healing assay on the AEY-TZP discs, both bergenin-treated groups showed a statistically significant increase in wound healing across both time points compared to untreated cells. Whilst an overall greater increase in healed percentage compared to control at 24 h was noted, it was not statistically significant. To explain the difference between the two data sets, further investigations into surface properties, material chemistry and surface characterisation are required. To the authors’ best knowledge, the effect of antiseptics or antibiotics commonly used in local delivery of a therapeutic agent in the management of peri-implant infections on the migration ability of HGF on zirconia surfaces remains unexplored. However, as noted previously, cellular proliferation was negatively affected as a result of the cytotoxic nature of other common antiseptics, thus implying the inability of cells to migrate in the presence of these antiseptics [24,25,26,29]. The current study was able to demonstrate an overall positive effect of bergenin on migration in the context of zirconia.

Although we have shown the non-cytotoxic nature of bergenin on HGF in the context of zirconia surfaces, it is important to note there are other materials that have comparable qualities that could also be considered suitable materials for implants. Polyetheretherketone (PEEK) has been shown to have comparable mechanical qualities to titanium but with a significant reduction in the ability of *Streptococcus oralis* to attach and form a biofilm [46]. Zirconia was not included in that study, but another study showed no significant difference between PEEK and zirconia in relation to biofilm-repelling activity [47]. They also showed that the cell viability of osteoblasts was comparable between PEEK and zirconia, so either material could be suitable as a dental implant material. Peng et al. (2021) did find surface roughness to be reduced in zirconia compared to PEEK, but this may be due to the choice of method for creating surface roughness [47]. Our studies have shown surface roughness of zirconia to be equivalent to that of titanium if sandblasting is used [8]. To avoid the overuse of antibiotics and antiseptics with potentially toxic effects on cells, we have proposed that bergenin, as a novel molecule with anti-microbial and anti-inflammatory activities, should be used as an adjunct therapy, but it should be recognised that photodynamic therapy is a viable alternative. Photodynamic therapy has been shown to have antimicrobial efficacy and to remove biofilm to a similar degree as antibiotics but the effect on cells in the surrounding tissue may still need to be evaluated [47,48]. Photodynamic therapy does generate reactive oxygen species that can lead to cellular destruction, so a direct comparison of photodynamic therapy and bergenin as an adjunct therapy would be valuable. More importantly, each patient needs to be evaluated for treatment as an individual, and it is useful to have a variety of options so that treatment can be tailored to their specific needs.

The results of this study showed the non-cytotoxic nature of bergenin on HGF in the context of zirconia surfaces. The HGF morphology, attachment, proliferation and migration of bergenin-treated cells were comparable to untreated cells. One key limitation of the present study was the use of a single cell type (HGF) as healing is a complex process involving a variety of cells and physiological mechanisms, such as inflammation. In addition, it is understood that surface modification can affect the cellular response of HGF; thus, identifying the surface characteristics could help establish a baseline and understand the extent of the effect of surface modification on cell response. Also, the potential for the phase transformation of the zirconia material due to its exposure to a high concentration of acid used in etching process will need to be acknowledged and explored further.

## 5. Conclusions

Within the limitations of an in vitro study, exposure to bergenin at both 2.5 and 5 µM concentration did not demonstrate a negative impact on the cellular characteristics and responses, including morphology, attachment, proliferation and migration, of human gingival fibroblasts. Additionally, investigations on the proposed anti-inflammatory and anti-microbial activity in the context of the oral environment is essential prior to the consideration of bergenin for the management of peri-implant inflammatory conditions clinically.

## Data Availability

The data presented in this study are available from the corresponding author upon reasonable request.
